# Does sleep aggravate tension-type headache?: An investigation using computerized ecological momentary assessment and actigraphy

**DOI:** 10.1186/1751-0759-5-10

**Published:** 2011-08-12

**Authors:** Hiroe Kikuchi, Kazuhiro Yoshiuchi, Yoshiharu Yamamoto, Gen Komaki, Akira Akabayashi

**Affiliations:** 1 Department of Psychosomatic Research, National Institute of Mental Health, National Center of Neurology and Psychiatry, 4-1-1 Ogawa-Higashi, Kodaira, Tokyo, 187-8553, Japan; 2 Department of Stress Sciences and Psychosomatic Medicine, Graduate School of Medicine, The University of Tokyo, 7-3-1 Hongo, Bunkyo-ku, Tokyo, 113-8655, Japan; 3 Educational Physiology Laboratory, Graduate School of Education, The University of Tokyo, 7-3-1 Hongo, Bunkyo-ku, Tokyo, 113-0033, Japan

## Abstract

**Background:**

Both insufficient sleep and oversleeping have been reported as precipitating and aggravating factors of tension-type headache (TTH). However, previous studies relied on recalled self-reports, and the relationship has not been confirmed prospectively and objectively in a daily life situation. Recently, ecological momentary assessment (EMA) using electronic diaries, i.e., computerized EMA, is used to record subjective symptoms with the advantages of avoiding recall bias and faked compliance in daily settings. In addition, actigraphy has become an established method to assess sleep outside laboratories. Therefore, the aim of this study was to investigate the within-individual effect of sleep on the following momentary headache intensity in TTH patients during their daily lives utilizing EMA and actigraphy.

**Methods:**

Twenty-seven patients with TTH wore watch-type computers as electronic diaries for seven consecutive days and recorded their momentary headache intensity using a visual analog scale of 0-100 approximately every six hours, on waking up, when going to bed, and at the time of headache exacerbations. They also recorded their self-report of sleep quality, hours of sleep and number of awakenings with the computers when they woke up. Physical activity was continuously recorded by an actigraph inside the watch-type computers. Activity data were analyzed by Cole's algorithm to obtain total sleep time, sleep efficiency, sleep latency, wake time after sleep onset and number of awakenings for each night. Multilevel modeling was used to test the effect of each subjective and objective sleep-related variable on momentary headache intensity on the following day.

**Results:**

Objectively measured total sleep time was significantly positively associated with momentary headache intensity on the following day, while self-reported sleep quality was significantly negatively associated with momentary headache intensity on the following day.

**Conclusions:**

Using computerized EMA and actigraphy, longer sleep and worse sleep quality were shown to be related to more intense headache intensity on within-individual basis and they may be precipitating or aggravating factors of TTH.

## Background

Tension-type headache (TTH) is a common pain disease with an estimated lifetime prevalence of 30-78% [1], and there have been studies discussing its characteristics in daily life, especially its relationship with psychological and behavioral factors. Regarding sleep, previous studies have reported that sleeping problems were associated with TTH, although the association might be explained by a comorbid mood or anxiety disorder [2-4]. In addition, both insufficient sleep and oversleeping, as well as sleep pattern changes, have been reported as precipitating or aggravating factors of TTH in cross-sectional studies using questionnaires or interviews [5-9]. Because those cross-sectional studies using questionnaires or interviews usually asked patients to list their precipitating or aggravating factors, they did not investigate actual relationships between headache and other factors directly but inquired about what patients thought made their headache occur or worse, i.e., patients' belief concerning precipitating and aggravating factors. No prospective studies or objective assessments of sleep have been performed in association with TTH.

Recently, ecological momentary assessment (EMA) has become a recommended method for investigating subjects' actual experiences, such as subjective symptoms, in their daily lives. In EMA, phenomena are assessed at the moment and at the place they occur in daily settings and its ecological validity is thought to be high because it is free of retrospective recall [10]. Although 'faked compliance' (i.e., disguise of compliance by recording data at times other than those designated) has been pointed out as a concern with EMA utilizing paper-and-pencil diaries, computerized EMA (i.e., EMA using computers as electronic diaries) has the advantage of avoiding faked compliance by recording the time at which data are inputted [11]. Because recall of pain has been reported to be affected by several factors [12-19], EMA is thought to be useful for investigating TTH. When investigating the relationship between TTH and other factors, computerized EMA is especially advantageous because faked compliance may be problematic when investigating temporal relationships. However, there have been few studies applying computerized EMA to TTH so far.

With regard to investigating sleep/wake patterns in daily settings, actigraphy has become an established method with its advantage over polysomnography that it is conveniently applied outside laboratories [20]. The combination of actigraphy and computerized EMA of momentary headache intensity would make it possible to know the actual relationship between sleep and TTH in daily settings.

Therefore, the aim of this study was to investigate the within-individual relationship between sleep and momentary headache intensity in TTH patients prospectively and objectively during their daily lives by utilizing actigraphy and computerized EMA, especially focusing on whether sleep affects headache intensity on the following day.

## Methods

All the procedures and materials were approved by the Ethics Committee of The University of Tokyo.

### Subjects

The subjects in the present study were a subgroup of those enrolled in another trial of relaxation therapy for TTH. Recruitment was conducted from March 2003 to August 2004 via an advertisement on the website of Department of Psychosomatic Medicine, The University of Tokyo, as well as on the websites of the clinic of neurology and the self-help group of chronic headache patients. Patients who applied for participation in this study were interviewed and screened by well-trained physicians, including the authors (HK and KY).

Inclusion criteria for the study were as follows: diagnosis of any type of TTH according to the criteria of the International Headache Society [21]; at least one TTH episode per week on average; and age ≥20 but <60 years old. Exclusion criteria were as follows: diagnosis of headache other than TTH according to the criteria of the International Headache Society [21]; current psychiatric disease; history of paranoia or schizophrenia; history of panic disorder, personality disorders; history of severe physical illnesses; current or prior participation in relaxation therapy; employment as a shift worker; and use of hypnotics. Eighty-four subjects applied to participate in the study and 59 met the eligibility criteria. Five declined participation due to scheduling conflicts, and therefore 54 were finally enrolled in the trial of relaxation therapy. Nineteen subjects who also had migraine, one subject who worked as a shift worker, and eight subjects who used hypnotics were excluded, leaving the data of 29 available for analysis. All the subjects gave their written informed consent to participation.

### Measurements

#### Measurement of momentary headache intensity

To record momentary headache intensity, watch-type computers (Ruputer ECOLOG; 42 grams, Seiko Instruments Inc., Tokyo, Japan) were used as electronic diaries [22]. The computer was equipped with a screen measuring 20 × 30 mm, and a joystick and a button as input devices. The subjects were fully instructed as to how to use the device and were given manuals before the beginning of the study period. They also practiced manipulating the device with one of the authors (HK) until they became accustomed to its use.

The subjects wore the watch-type computers for seven consecutive days. Signal-contingent recordings were defined as recordings that were prompted with a beep as a signal [10] and they were programmed to occur randomly within an interval of 36 minutes around 6:00, 12:00, 18:00, and 24:00. If the subjects did not enter a recording when the computer beeped, they were allowed to postpone input for 30 minutes. Recordings not made within 30 minutes were cancelled. The subjects were also asked to record their headache intensities when they woke up and went to bed by choosing 'waking up' or 'going to bed' from the menu. After selecting a 'going to bed' recording, computers suspended signal-contingent recordings until a 'waking up' recording was selected to avoid disturbance of sleep. Signal-contingent recordings and recordings when waking up and going to bed were treated as scheduled recordings.

Event-contingent recordings were defined as recordings initiated by the subjects themselves when a particular event occurred [10]. In the present study, the subjects were asked to make a recording every time their headache became exacerbated, with or without taking analgesics, as an event-contingent recording.

In both scheduled and event-contingent recordings, momentary headache intensity was rated according to a visual analog scale from 0 to 100 displayed on the screen. The words "headache intensity" were displayed with the visual analog scale as a question, and the anchor words "none" and "most intense" were displayed at the respective ends of the scale. By manipulating the joystick, the subjects adjusted the length of the bar so that it corresponded to their headache intensity at that moment. In recordings when waking up, self-report of sleep quality, hours of sleep and number of awakenings was also recorded. Sleep quality was rated according to the same visual analog scale accompanied by "not at all" and "most" at both ends and "How much have you been refreshed?" as a question. Hours of sleep and number of awakenings were recorded by selecting from the pull-down menu displayed on the screen. The pull-down menu for hours of sleep ranged from "less than four hours" to "more than or equal to nine hours" and it was divided on the hourly basis between them. The pull-down menu for number of awakenings consisted of 8 items from "none" to "more than or equal to 7".

#### Measurement of sleep using actigraph

Wrist activity monitors were built into the watch-type computers used for recording momentary headache intensity and were worn all day and night on the non-dominant hand. The subjects wore them for seven consecutive days as mentioned above. The instrument was removed for bathing, showering, or any other activity for which water damage was likely. The time the instrument was taken off and put back on was recorded when 'taking off' or 'putting on' item was selected from the menu. The wrist activity monitors are uni-axial piezo-electronic accelerometers with a sensitivity of 0.01 g, and are analogous in performance to the Actigraph Mini-Motionlogger (Ambulatory Monitors Inc., Ardsley, NY), which has frequently been used in studies of physical activity. Zero-crossing mode was used and acceleration counts were accumulated for every epoch of one minute.

### Data analysis

Activity data were analyzed by Cole's algorithm [23] and the following sleep parameters were calculated; total sleep time (time spent asleep after sleep onset), sleep efficiency (total time asleep as a percentage of total time from 'going to bed' to 'waking up'), sleep latency (time between 'going to bed' and sleep onset), wake time after sleep onset (WASO, time spent awake from sleep onset to final awakening), number of awakenings (number of blocks of contiguous wake epochs between sleep onset to 'waking up').

### Statistical analysis

Firstly, we employed multilevel modeling to estimate a grand mean (mean of individual mean) between-individual variance and within-individual variance of momentary headache intensity and sleep parameters instead of showing their descriptive statistics because the dataset in the present study was hierarchical; momentary recordings were nested within individuals. Multilevel modeling was also used to compare objectively measured total sleep time and self-reported hours of sleep before analyzing their relationships with headache. Secondly, multilevel modeling was used to investigate within-individual relationships between momentary headache intensities and sleep. Momentary headache intensity was used as a dependent variable and each sleep parameter measured by actigraph for the previous night was used as the predictor in separate models. The effect of sleep parameters was modeled either as a fixed effect or a random effect. In order to control for the time of momentary headache recording, time of day was divided into four blocks (3:00-9:00, 9:00-15:00, 15:00-21:00 and 21:00-3:00; with centers corresponding to the times of signal-contingent recordings) and entered into a model as covariates, if necessary. The following models were tested for each sleep parameter: model 1, the unconditional model (no predictor); model 2, an unconditional model with control for time; model 3, a model with the effect of the sleep parameter as a fixed effect; model 4, a model with the effect of the sleep parameter as a random effect; model 5, a model with the effect of the sleep parameter as a fixed effect and with control for time; model 6, a model with the effect of the sleep parameter as a random effect and with control for time. The level 1 intercept (individual true value of momentary headache intensity when the predictor and covariates were zero) was modeled as a random effect. Variance-covariance matrix (G matrix) was modeled as unstructured except in cases of nonconvergence where it was instead modeled as 'variance components', in which all the covariances were modeled as zero. When the effect of the sleep parameter was significant, percent reduction of the residual variance from the unconditional model was calculated to determine how much of the variance of momentary headache intensity could be explained by the sleep parameter. Self-reported sleep quality, hours of sleep and number of awakenings were analyzed in the same way, with sleep parameters measured by actigraph. Number of awakenings was treated as a continuous variable and self-reported hours of sleep was treated both as a continuous variable and a categorical variable.

In all analyses, goodness of fit was compared using -2 log likelihood function and χ^2 ^test when one model was nested in the other; otherwise Akaike's Information Criterion was used. All analyses were conducted with SAS Proc Mixed (SAS 9.1, SAS Institute Inc., Cary, NC).

## Results

### Patient characteristics

Twenty-nine subjects were enrolled and two were excluded from further analyses because they were unable to complete their recordings for seven days due to problems with the computers. Finally, 27 subjects (seven men and 20 women) were analyzed and their profiles are shown in Table 1. The mean age of the subjects was 37.4 years old (SD, 9.4 years; range, 25-59 years old). Five subjects had episodic tension-type headache (i.e., the number of days with headache < 15/month), 20 had chronic tension-type headache (i.e., the number of days with headache ≥ 15/month), and two had headache of tension-type not fulfilling the criteria of episodic or chronic tension-type headache. Nine subjects regularly took prophylactic medication and 13 used analgesics on-demand.

**Table 1:** Demographic and medical characteristics of the subjects

Sex	
Women	20(74%)
Men	7(26%)
	
Age	
Mean in years (SD)	37.4(9.4)
	
Subtype of tension-type headache	
Episodic	5 (19%)
Chronic	20 (74%)
Other	2 (7%)
	
Prophylactic medication	
With	7(26%)
Without	20 (74%)
	
Use of on-demand medication	
With	13 (48%)
Without	14 (52%)

SD, standard deviation.

### Recording profiles

For all subjects, there were 878 scheduled recordings consisting of 502 signal-contingent recordings, 187 recordings on awakening, and 189 recordings at bedtime. The mean compliance rate for signal contingent recordings was 97.5%. The mean number of scheduled recordings was 32.5 per subject. Nineteen subjects made 81 event-contingent recordings and the other eight subjects made no event-contingent recordings.

### Grand means, between- and within-individual variances of momentary headache intensity and sleep parameters

Table 2 shows grand means, between- and within-individual variances of momentary headache intensity and sleep parameters estimated by multilevel models.

**Table 2:** Estimated grand mean and between- and within-individual variance of momentary headache intensity and sleep parameters

	Grand mean (SE)	Between-individual variance (SE)	Within-individual variance (SE)
Momentary headache intensity (0-100)	36.4(4.1)	449.3(124.9)	334.4(15.6)
Parameters from actigraphic data			
Total sleep time (min)	412.2(12.3)	2800.2(1116.6)	8407.3(961.0)
Sleep efficiency (%)	90.5(1.3)	41.4(13.1)	45.8(5.3)
Sleep latency (min)	25.6(4.3)	400.9(136.2)	679.0(78.2)
WASO (min)	19.5(3.4)	242.8(84.8)	463.4(52.9)
Number of awakenings	2.59(0.33)	2.04 (0.79)	5.28(0.61)
Parameters from self-reports			
Sleep quality (0-100)	44.4 (2.4)	124.3 (42.7)	209.3 (23.9)
Hours of sleep (hour)	6.40 (0.19)	0.80 (0.27)	1.20 (0.15)
Number of awakenings	1.22 (0.17)	0.64 (0.21)	0.85 (0.10)

SE, standard error.

WASO, wake time after sleep onset.

### Comparison between objectively measured total sleep time and self-reported hours of sleep

There was a significant association between objectively measured total sleep time and self-reported hours of sleep, which was shown by the model with objectively measured total sleep time as a dependent variable and self-reported hours of sleep (converted into the central value of the interval in units of minutes) as an independent variable; the estimated coefficient of self-reported hours of sleep was 1.02 (standard error = 0.07, p < 0.0001) and the estimate of intercept was 18.0 (standard error = 29.7, p = 0.55). However, the difference between them significantly differed from zero; the estimated difference was 27.2 (standard error = 13.0, p = 0.046).

### Effect of objectively measured sleep parameters on headache intensity on the following day

For sleep efficiency, sleep latency, WASO and number of awakenings, no models with the effect of a sleep parameter (model 3-6) had better fit than the unconditional models. For total sleep time, the model with the effect of total sleep time as a fixed effect and with control for time (model 5) had better fit than the other models and the effect of total sleep time was significant in model 5 (coefficient = 0.027, F(1, 796) = 10.11, p = 0.015; Table 3). Percent reduction of the residual variance was 2.6%. Model 5 was expressed as follows:

**Table 3:** Effects of objective and self-reported sleep parameters on headache intensity on the following day

	Objective measure		Self-report	
		
	Total sleep time		Sleep quality	
		
	Coefficient (SE)	p value	Coefficient (SE)	p value
Intercept	29.7 (5.4)		47.3 (4.9)	
Effect of sleep parameter	0.027 (0.008)	p = 0.015	-0.14 (0.05)	p = 0.008
Control for time		p < 0.0001		p < 0.0001

SE, standard error.

Intercept is estimated mean headache intensity when the sleep parameter is zero and time is in 15:00-21:00.

Level 1 equation:

Level 2 equations:

where Y*_ij _*is each momentary headache intensity for the *i*th patient; SLEEP*_ij _*is the corresponding total sleep time; and TIME0*_ij_*, TIME6*_ij _*and TIME12*_ij _*are dummy variables indicating that the time of day was 21:00-3:00, 3:00-9:00 and 9:00-15:00, respectively (e.g. TIME0*_ij _*= 1 if the time of day was in 21:00-3:00, TIME0*_ij _*= 0 otherwise). π_0*i *_is the individual *i*'s true value of headache intensity when all predictors are zero (intercept). γ_00 _is the average true value of mean headache intensity when all predictors are zero. π_1*i *_is the individual *i*'s slope representing the effect of total sleep time on momentary headache intensity and γ_10 _is the average slope. ε*_ij _*and ζ_0*i *_are residuals at each level. Including ζ_0*i *_in the equation means that the intercept was modeled as random, which suggested that intercept could vary across individuals. The second level 2 equation include no residual, which means that the effect of total sleep time on momentary headache intensity was modeled as a fixed effect in model 5. π_2*i*_, π_3*i *_and π_4*i *_are the individual *i*'s true differences in momentary headache intensity between corresponding time blocks and the reference time block. They are equal to γ_20_, γ_30 _and γ_40_, which are the average differences, because the effect of time was modeled as a fixed effect.

**Figure 1:**
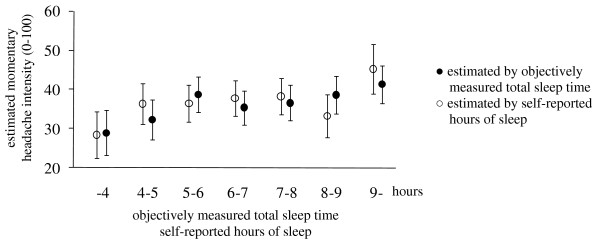
**Momentary headache intensity estimated by multilevel modeling with either objectively measured or self-reported sleep time**. Estimates are shown with standard error as error bars. Filled circles are estimates by the model with objectively measured total sleep time and open circles are estimates by the model with self-reported hours of sleep. Time of day was controlled and estimates of when time is in the reference block (15:00-21:00) are shown.

Figure 1 shows estimated headache intensity from the model with total sleep time as a categorical variable after converting it using the same class intervals for self-reported hours of sleep.

### Effect of self-reported sleep parameters on headache intensity on the following day

For self-reported number of awakenings and hours of sleep, no models with their effect (model 3-6) had better fit than the unconditional models. For self-reported sleep quality, the model with the effect of sleep quality and with control for time (model 5) had better fit than the other models and the effect of sleep quality was significant in model 5 (coefficient = -0.14, F(1, 761) = 6.99, p < 0.008; Table 3). Percent reduction of the residual variance was 0.88%. Estimated headache intensity from the model with self-reported hours of sleep was shown in Figure 1.

## Discussion

The relationship between objective assessment of sleep and momentary headache intensity in the daily lives of TTH patients was investigated prospectively using actigraphy and computerized EMA, making this the first study of sleep and TTH using this approach.

Total sleep time measured by actigraph was significantly and positively associated with headache intensity on the following day, suggesting that TTH patients experienced more intense headache when they slept longer on the previous night. In other words, oversleep might possibly be precipitating or aggravating factors of TTH and may support a result of a previous study in which 12.9% of TTH patients reported that oversleep was a precipitating factor of TTH [5]. On the contrary to this result, previous cross-sectional studies based on questionnaires and interview also showed that many TTH patients reported that insufficient sleep was a precipitating or aggravating factor of TTH [5,6,8]. This inconsistency might be because those previous studies using questionnaires and interviews actually do not investigate the real relationship between TTH and sleep itself, but asked patients' belief about their TTH, i.e. what patients thought about their TTH and its associations with sleep, which might be true to the facts or a wrong assumption unrelated to the facts. To test the hypothesis that both insufficient sleep and oversleeping are associated with intense headache, models including the quadratic term of total sleep time could be used. However, they are difficult to test using weeklong data because there was not a sufficient number of nights and the estimated headache intensity by the model with total sleep time as a categorical variable (Figure 1) did not seem to support that model with the quadratic term. There is also the possibility that precipitating or aggravating factors are different among patients; however, the fact that the model with the effect of total sleep time as a fixed effect had better fit than the model with it as a random effect suggested that the effect of total sleep time on headache intensity did not significantly differ among patients.

There was no significant association between self-reported hours of sleep and momentary headache intensity on the following day, which was inconsistent with the result of total sleep time. There are some studies that compare objective sleep parameters (measured by polysomnography or actigraph) and self-reported sleep parameters in various populations such as healthy subjects [24], patients with depression [25,26], insominia [27] and chronic low back pain [28]. The difference between objective and subjective parameters was often pointed out and it has been suggested that both parameters should be applied. In this study, although objectively measured total sleep time and self-reported reported hours of sleep were significantly associated on a within-individual basis, self-reported hours of sleep were significantly shorter than objectively measured total sleep time. The inconsistent results of the self-reported hours of sleep could be interpreted by assuming that evaluation by actigraph is reliable and self-reports are unreliable because the subject can not accurately recall how many hours they have slept. Recently, it has been pointed out that when people are not able to recall their experiences, they make their self-report based on their belief, making up for lack of memories [29]. Therefore, the result of the self-reported sleep might be a consequence of systematic bias reflecting the patients' belief that "insufficient sleep leads to intense headache", which were somehow inconsistent with objectively measured sleep. When their momentary headache is intense in the morning, they might have thought that sleep might have been short based on their belief and recorded their self-reported hours of sleep shorter than the real hours of sleep. This bias might have attenuated the actual relationship.

Self-reported sleep quality was also shown to be associated with momentary headache intensity. Sleep quality is a fairly subjective concept and no objective measure of sleep quality has been established yet [30]. Therefore, it is not possible to know if any physiological process underlies the association of self-reported sleep quality with momentary headache intensity. There is a possibility that feeling that they slept well itself may have an effect which makes their headache better. It was also pointed out that ratings of sleep quality may reflect factors other than sleep, such as mood states [30]. In this study, there is also the possibility that headache intensity may affect the ratings of sleep quality.

There are some limitations in the present study. Firstly, one week may not be long enough for investigating within-individual relationships between headache intensity and sleep. This might lessen the generalizability of the results of this study. Secondly, the effect of sleep on headache intensity on the following day was analyzed in the present study, whereas sleep over several preceding nights might affect headache intensity. Headache might worsen with insufficient sleep for several days. Longer observation times and application of variables processed in other ways may provide more information on the relationship between sleep and headache intensity in TTH. Thirdly, percent reduction of residual variance was small for both sleep measured by actigraph and self-reported sleep. This means that only a small part of the headache variation could be explained by sleep. In addition, even though the effects of sleep-related variables were significant, their coefficients are generally rather small. Therefore, it is not clear whether or not these statistically significant relationships are clinically significant.

## Conclusions

Using actigraphy and computerized EMA, the relationship between headache intensity and sleep in the daily lives of TTH patients was demonstrated, and the results suggested that a longer total sleep time and worse sleep quality might be precipitating or aggravating factors of TTH. Further comprehensive investigations with longer observation periods, more detailed information about their beliefs, and as well as other physiological measures such as polysomnography would be helpful for better understanding sleep and TTH.

## List of abbreviations

TTH: tension-type headache; EMA: ecological momentary assessment; WASO: wake time after sleep onset.

## Competing interests

The authors declare that they have no competing interests.

## Authors' contributions

HK designed the study, collected the data, analyzed the data, performed the statistical analysis, interpreted the results, and drafted the manuscript. KY designed the study, interpreted the results, and drafted the manuscript. YY, GK and AA helped interpret the results, and draft the manuscript. All authors read and approved the final manuscript.
